# Simulation and Reproduction of Direct Solar Radiation Utilizing Grating Anomalous Dispersion

**DOI:** 10.3390/s25247474

**Published:** 2025-12-09

**Authors:** Junjie Yang, Jian Zhang, Bin Zhao, Lu Wang, Yu Zhang, Songzhou Yang, Da Xu, Taiyang Ren, Jingrui Sun, Guoyu Zhang

**Affiliations:** 1 School of Optoelectronic Engineering, Changchun University of Science and Technology, Changchun 130022, China; 2 Jilin Optoelectronic Measurement and Control Instrument Engineering Technology Research Center, Changchun 130022, China; 3 Key Laboratory of Optoelectronic Measurement and Control and Optical Information Transmission Technology of Ministry of Education, Changchun 130022, China; 4 State Key Laboratory of High Power Semiconductor Laser, Changchun University of Science and Technology, Changchun 130022, China; 5 School of Artificial Intelligence, Changchun University of Science and Technology, Changchun 130022, China

**Keywords:** solar simulator, grating anomalous dispersion, direct solar radiation simulation, solar angular diameter simulation, spectral shape reconstruction, beam reconstruction

## Abstract

The technical challenge of balancing radiant illuminance and the angular diameter of the simulated sun remains unsolved, preventing the realization of a solar simulator with both a 32′ angular diameter and a solar constant irradiance. This paper proposes a direct solar radiation simulation method using grating anomalous dispersion and a technological implementation scheme. This new architecture consists of a spectrally modulated optical engine, a diffractive combining system, and a multi-aperture imaging reconstruction system. We designed an optical system for simulating direct solar radiation, which achieves a high degree of reproducibility of natural direct solar radiation characteristics. The performance of this system was verified through simulation, with the results indicating that the solar direct radiation simulator achieves an angular diameter of 31.7′ while maintaining radiant illuminance above a solar constant. Additionally, the system spectral match to both the extraterrestrial (AM0G) and terrestrial global (AM1.5G) solar spectra, along with its uniformity, complies with an A+ grade. The studied direct solar radiation simulation is currently the only instrument capable of achieving a solar constant of an angular diameter less than 32′. This research revolutionizes the structure and principle of the traditional solar simulator, makes up for the deficiencies of the existing solar simulation technology, further improves the theoretical system of solar direct radiation simulation, and has far-reaching scientific significance for the development and application of solar simulation technology.

## 1. Introduction

Direct solar radiation is essential in fields such as aerospace [1], meteorological and environmental science [2], and solar energy conversion, storage, and application [3]. However, its application in nature is hampered by unpredictable meteorological conditions and intermittency, rendering it challenging to ensure stability in test environments that utilize direct solar radiation [4,5]. Consequently, solar simulators, which provide controllable, reliable, and reproducible indoor characterization experimental environments, have become crucial research tools.

Solar simulators are always significantly affected by the mutual constraints between radiant illuminance and angular diameter [6,7], and have not been able to realize the simulation of a solar constant at an angular diameter of 32′ [8]. The use of advanced optical design techniques, such as Monte Carlo ray-tracing analysis, has spurred active research in improving solar simulator [9] designs and performance. The discharge lamp, known for its high radiant power and spectral characteristics close to the natural solar spectrum, has become a popular choice [10,11]. In 2019, Jin et al. developed a universal solar simulator with a xenon lamp [12], achieving an average radiant illuminance of 940 W/m^2^, an angular diameter of 2.6°, and 8% non-uniformity across a 4000 mm × 3000 mm area. However, the study did not correct the spectrum of the xenon lamp, resulting in a discrepancy from the natural solar spectrum [13]. In 2020, Zhu et al. constructed a solar simulator using direct multiple lamps mapping [14] and achieved an average radiant illuminance exceeding 1000 solar constants over a 200 mm diameter area and 18.59% non-uniformity over a 60 mm diameter area. Despite these enhancements, the simulator still lacked solar spectral matching and angular diameter considerations. In order to improve spectral matching, in 2021, Martínez-Manuel et al. designed a solar simulator using a metal halide [15] lamp, which more closely matched the spectral distribution of AM1.5G compared with previous xenon lamp-based simulators [16]. This simulator achieved 1198 W/m^2^ over a 2000 mm × 1000 mm target area, with an average radiant illuminance and 1.4% non-uniformity over the same area. Nonetheless, this design also did not adequately address the constraints on the solar angular diameter or spectrum matching.

With the increased efficiency of photovoltaic (PV) devices, innovation in materials, iteration of product technologies, and reduction in costs [17,18], a growing demand for improved spectral matching [19,20] and angular diameter [21,22] in solar simulators exists. Concurrently, advancements in the spectral coverage [23] and energy efficiency [24] of LED light sources have shifted the focus toward LED solar simulators that offer superior solar spectral matching and closer to the 32′ angular diameter [25]. In 2021, Al-Ahmad et al. introduced an economical LED solar simulator [26], achieving spectral matching with AM1.5G from 350 nm to 1100 nm, and attaining Class A in terms of non-uniformity and stability. They later developed a large-scale LED solar simulator [27] that exhibited only 1.7% non-uniformity over an irradiated area of 320 mm × 200 mm and met Class A spectral matching standards. However, these developments overlooked the constraints on the angular diameter. In response to this limitation, in 2022, Sun et al. designed a solar simulator based on high-power narrow-band LEDs [28], which achieved Class AAA simulation with radiant illuminance equivalent to a solar constant over an irradiated area of 50 mm × 50 mm (AM1.5G solar spectrum). Despite its advancements, the divergence angle of the collimated beam was ±3°, significantly differing from the true solar angular diameter. In 2023, Du et al. developed a large-area hybrid solar simulator [29], which achieved a correlation coefficient of 0.90 between the measured and reference spectra within the range of 350–2500 nm and provided average radiant illuminance of 762 W/m^2^ and 274 W/m^2^ at solar zenith angles of 10° and 70°, respectively, across a 1000 mm × 1000 mm area.

The 32′ solar angular diameter represents the true geometric characteristics of the sun in the sky, and its accurate simulation is critical for practical solar simulator applications. In aerospace applications, sun sensors determine satellite attitude by measuring the solar vector direction. Accurate simulation of the 32′ solar angular diameter can reduce angle-induced errors in sun sensor measurements, thereby resolving the testing accuracy limitations resulting from inadequate angular precision in current systems. In photovoltaic testing, the simulation of solar angular diameter directly affects the precision of photovoltaic performance evaluation at different incidence angles. The current simulator’s angular diameter simulation accuracy no longer meets high-precision testing requirements. Therefore, accurately reproducing the 32′ solar angular diameter is both a major technological breakthrough and the key to enhancing the practical value of solar simulators.

These developments indicate a progression in solar simulator sources from xenon lamps to metal halides, and now to LED arrays. This evolution includes transitioning from single-lamp to multi-lamp illumination, and from optical integrators to stacked multi-lamp illumination, achieving significant advances in radiant illuminance, angular diameter, non-uniformity, and spectral matching. Nonetheless, no solar simulator yet meets the standards of a solar constant under a 32′ angular diameter while simultaneously simulating AM0G and AM1.5G.

To address these challenges, this paper proposes a novel approach to solar radiation simulation and reproduction using grating anomalous dispersion and constructs a new architecture consisting of a spectrally modulated optical engine, diffraction combining system, and multi-aperture imaging reconstruction system. This paper diverges from traditional methods, such as high-power mirrored light sources or direct LED superposition, which can effectively solve the technical challenge of mutual constraints between radiant illuminance and angular diameter that has been plaguing the research field of solar simulators, and realize the accurate simulation and reproduction of indoor natural direct solar radiation.

## 2. Principles of Direct Solar Radiation Simulation and Reproduction

### 2.1. Overall Architecture of Direct Solar Radiation Simulator Utilizing Grating Anomalous Dispersion

**Figure 1:**
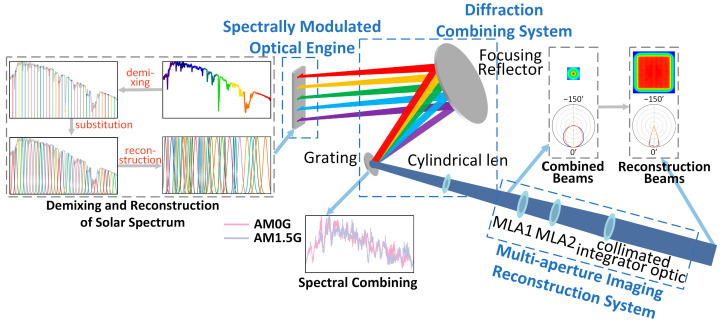
Overall architecture and simulation principle of the solar direct radiation simulator utilizing grating anomalous dispersion.

The overall architecture of the solar direct radiation simulator, which utilizes grating anomalous dispersion, comprises three main components: the spectrally modulated optical engine, diffraction combining system, and multi-aperture imaging reconstruction system, as shown in Figure 1.

The spectrally modulated optical engine utilizes various LED-based light sources to emit light beams, which are converged by focusing mirrors within the diffraction combining system. These beams are directed to the grating on the focal plane of the focusing mirror. The grating here plays the role of anomalous dispersion, because light of different wavelengths passing through the grating will produce a diffraction angle related to the wavelength. By designing the incidence angle of each narrowband LED sub-beam, the grating causes light of different wavelengths to converge in the same outgoing direction after diffraction, enabling spectral beam combining. The combined beams are then projected onto a cylindrical lens, which minimizes the disparity between the beams in the meridian and sagittal directions, matching their angular divergence to the receiver field angle of the multi-aperture imaging reconstruction system, before being coupled into the system. Here, the first microlens array (MLA1) divides the beams, which are subsequently collimated by the second microlens array (MLA2) and a collimated integrator optic. This segmented and collimated beam simulates direct solar radiation as if emanating from infinity.

The research on simulating and reproducing direct solar radiation in this paper is structured into two distinct parts:

1. An optical model for solar spectral reconstruction based on anomalous dispersion is established. This model defines the precise mapping relationship between different peak wavelengths of narrowband LEDs and their positions within the spectral modulation optical engine. The diffraction combining system is subsequently utilized to control the emission angle and spot overlap of each narrowband LED beam after spectral combining.
2. An optical model for beam reconstruction is developed, and the diffracted energy distribution of the reconstructed beam is numerically simulated. This model determines the influence of the number of one-dimensional channels in the multi-aperture imaging reconstruction system on the non-uniformity of the reconstructed beam, both for single-wavelength and composite spectra. This leads to the design of the multi-aperture imaging reconstruction system that aims to meet the constraints on the non-uniformity and angular diameter of the solar simulator.

### 2.2. Solar Spectral Reconstruction

The solar spectrum is a continuous spectrum that spans a broad range of wavelengths and is rich in spectral details [30]. To effectively match the solar spectrum, initially, demixing the continuous spectrum into discrete spectral units is necessary. These units are represented by the peak wavelengths of narrow-band LEDs. Subsequently, the grating anomalous dispersion effect is employed to reconstruct the solar spectrum.

**Figure 2:**
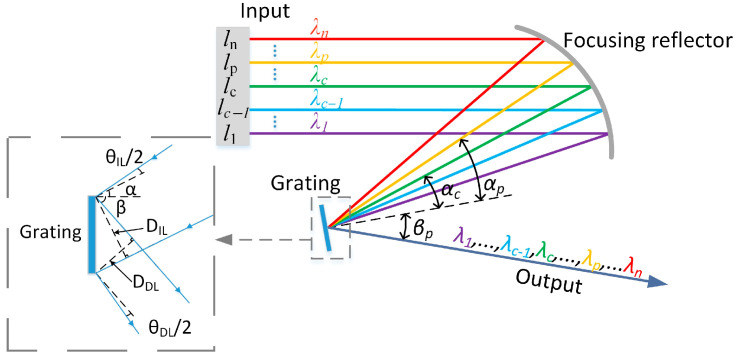
Optical principle of solar spectrum reconstruction based on anomalous dispersion.

Solar spectral reconstruction involves the scalar superposition of spectral reconstruction units. This process can effectively utilize the anomalous dispersion properties of gratings to mix and combine beams emitted from narrow-band LEDs with different peak wavelengths. This method offers significant advantages, including controllable emission angles after spectral combining and high energy utilization. These benefits are notable when compared with other spectral combining methods, such as integrating spheres and integrating rods, which rely on the diffuse reflection effect [31]. The optical principle of solar spectrum reconstruction based on anomalous dispersion is illustrated in Figure 2.

The narrowband LED with peak wavelength $\lambda_{c}$ in Figure 2 is located at the center of the spectral modulation optical engine, and $\alpha_{c}$ and $\beta_{c}$ are the angles of incidence and diffraction of this narrowband LED on the grating, respectively; if the peak wavelength spacing between neighboring narrowband LEDs is Δλ, the peak wavelength of the narrowband LED in the pth spectral reconstruction unit is $\lambda_{p}=\lambda_{c}+(p-c)\Delta \lambda$, and its angle of incidence is $\alpha_{p}$ and diffraction angle is $\beta_{p}$. All narrowband LEDs are governed by the grating equation, which can be articulated as follows:

(1) $$d(\sin\alpha_{p}-\sin\beta_{p})=m\;\lambda_{p}$$

where d is the grating constant and m is the number of diffraction stages. At this point, let the focal length of the focusing reflector be $f_{FR}$. We differentiate both sides of Equation (1). Accordingly, the coordinates $l_{P}$ of the pth narrowband LED relative to the center position are

(2) $$l_{p}=f_{FR}\frac{m}{d\cos\alpha_{c}}(p-c)\Delta \lambda$$

Since all beams are output in the same direction after diffraction, $\beta_{p}=\beta_{c}$, at this point, $\alpha_{p}$ can be expressed as

(3) $$\alpha_{p}=\arcsin(\sin\alpha_{c}-m(p-c)\Delta \lambda )$$

Substituting Equation (2) into Equation (3), the relationship between the angle of incidence $\alpha_{p}$ of the pth narrowband LED at the grating and its coordinate $l_{P}$ is

(4) $$\alpha_{p}=\arcsin(\sqrt{1-(f_{FR}\frac{m}{dl_{p}}(p-c)\Delta \lambda {)}^{2}}-m(p-c)\Delta \lambda )$$

The narrowband LED possesses a specific spectral width and physical size, which necessitates the introduction of its beam diameter $D_{IL}$ and beam collimation angle $\theta_{IL}$ before diffraction to analyze changes during the diffraction process. This analysis aids in precisely controlling the beam diameter $D_{DL}$ and beam collimation angle $\theta_{DL}$ after diffraction. Consequently, a model depicting the variations in the narrowband LED before and after diffraction has been established, as illustrated in Figure 2. This model demonstrates that

(5) $$D_{DL}=D_{IL}\frac{\cos\beta }{\cos\alpha }$$

According to the law of conservation of optical expansion, $\frac{\theta_{\mathrm{DL}}}{\theta_{\mathrm{IL}}}=\frac{\cos\alpha }{\cos\beta }$. $\lambda_{FWHM}$ is the peak half-width of the narrowband LED spectrum; thus, the divergence angle of the narrowband LED is

(6) $$\theta_{\mathrm{DL}}=\frac{\cos\alpha }{\cos\beta }\theta_{\mathrm{IL}}+\frac{m}{2\mathrm{dcos}\beta }\lambda_{FWHM}$$

### 2.3. Beam Reconstruction Methods

#### 2.3.1. Optical Principle

**Figure 3:**
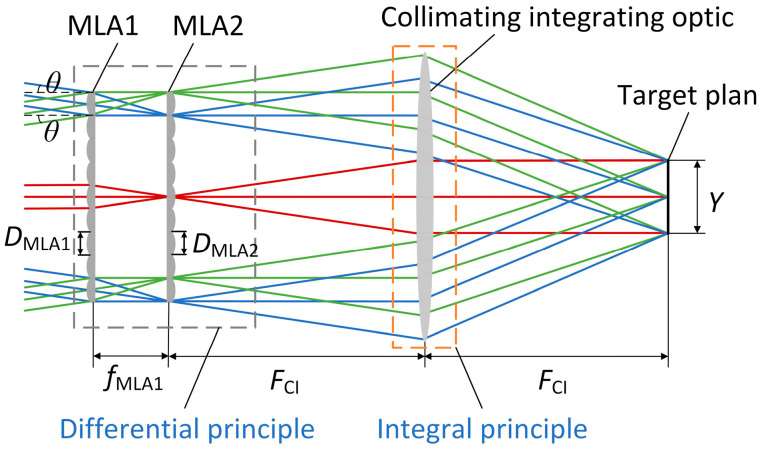
Optical principle of beam reconstruction.

The multi-aperture imaging-based reconstruction system comprises two microlens arrays and a collimated integrating mirror. The first microlens array segments the beam processed by the diffractive combining system into multiple sub-beams, each incident into different channels. The second microlens array, in conjunction with the collimated integrating optic, superimposes the beams from each channel of the first microlens array onto the target surface. The optical principle of beam reconstruction is depicted in Figure 3.

In Figure 3, the distance between the two microlens arrays is set equal to the focal length of the first microlens array [32]. The maximum half angular field of view θ of the incident beam in the multi-aperture imaging-based reconstruction system and the spot size, Y, on the target surface are

(7) $$\theta =\arctan\frac{D_{MLA2}}{2\;f_{MLA1}}$$

(8) $$Y=\frac{F_{CI}}{f_{MLA2}/D_{MLA1}}$$

where $f_{MLA1}$ and $f_{MLA2}$ denote the focal lengths of the sublenses in the two microlens arrays, respectively, and $D_{MLA1}$ and $D_{MLA2}$ denote the diameters of the sublenses in the two microlens arrays, respectively. The focal lengths and diameters of the sublenses in the two microlens arrays are equal, i.e., $f_{MLA1}=f_{MLA2}=f_{MLA}$, $D_{MLA1}=D_{MLA2}=D_{MLA}$, and $F_{CI}$ is the focal length of the collimating integrating mirror.

#### 2.3.2. Reconstructing Beam Intensity Distribution

The beam diffractively propagates through free space, modulated by the microlens array, and ultimately forms a specific diffracted energy distribution on the target surface. Assuming no absorption by the material, a microlens array with a 100% fill rate can be considered a phase modulation element [33]. The transfer function T(y) of the microlens array can be treated as the convolution of the transfer function of a single microlens unit with the comb function. This paper focuses on the square aperture lens array composed of 2*n* + 1 microlenses, thereby necessitating consideration only of the one-dimensional case, T(y). At this juncture, the transfer function is expressed as follows:

(9) $$T(y)=\sum_{n=-N}^{N}\delta (y-nD_{MLA})⊗\left[rect(\frac{y}{D_{MLA}})\times \exp(-ik\frac{y^{2}}{2f_{MLA}})\right]$$

where the comb function $\sum_{n=-N}^{N}\delta (y-nD_{MLA})$ represents the arrangement of the microlens array in the one-dimensional direction, $rect(\frac{y}{D_{MLA}})$ is a rectangular function that describes the sub-lens aperture, and $\exp(-ik\frac{y^{2}}{2f_{MLA}})$ represents the quadratic phase modulation introduced by the microlens array, at which time a monochromatic plane wave of wavelength $\lambda_{i}$ forms an amplitude distribution $U(y_{H})$ on the target surface:

(10) $$\begin{array}{c} U(y_{H})=\frac{\exp(ikF_{CI})}{ikF_{CI}}\int_{-\infty }^{\infty }\frac{\exp(ikF_{CI})}{ikF_{CI}}\int_{-\infty }^{\infty }\frac{\exp(ikf_{MLA})}{ikf_{MLA}}\int_{-\infty }^{\infty }U_{0}(y_{1})\times T(y_{1})\times \exp\left[ik\frac{{\left(y_{2}-y_{1}\right)}^{2}}{2f_{MLA}}\right]dy_{1}\\ \times T(y_{2})\times \exp\left[ik\frac{{\left(y_{F}-y_{2}\right)}^{2}}{2F_{CI}}\right]dy_{2}\times \exp(-ik\frac{y_{F}^{2}}{2F_{CI}})\times \exp\left[ik\frac{{\left(y_{H}-y_{F}\right)}^{2}}{2F_{CI}}\right]dy_{F} \end{array}$$

where $y_{1},y_{2},y_{F},y_{H}$ denote the positional coordinates of the first microlens array plane, second microlens array plane, collimated integrating optic plane, and target surface, respectively.

The intensity distribution $I(y_{H})$ on the target plane is

(11) $$I(y_{H})={\left|U(y_{H})\right|}^{2}\propto \left|\sum_{m=-\infty }^{\infty }\delta (y_{H}-m\frac{\lambda_{i}F_{CI}}{D_{MLA}})\right.\cdot {\left.\left[\sin c(\frac{D_{MLA}}{\lambda_{i}F_{CI}}y_{H})⊗rect(\frac{f_{MLA}}{F_{CI}D_{MLA}}y_{H})\right]\right|}^{2}$$

Considering that the solar spectrum is a complex color light, the intensity distribution $I_{solar}(y_{H})$ in the solar spectral range on the target surface is

(12) $$I_{solar}(y_{H})=\propto \int_{\lambda_{1}}^{\lambda_{n}}S_{solar}(\lambda_{i})\cdot \left|\sum_{m=-\infty }^{\infty }\delta (y_{H}-m\frac{\lambda_{i}F_{CI}}{D_{MLA}})\right.\cdot {\left.\left[\sin c(\frac{D_{MLA}}{\lambda_{i}F_{CI}}y_{H})⊗rect(\frac{f_{MLA}}{F_{CI}D_{MLA}}y_{H})\right]\right|}^{2}d\lambda_{i}$$

## 3. Optical System Analysis and Optimization for Direct Solar Radiation Simulation

### 3.1. Diffraction Beam Combining System Optimization Design

**Figure 4:**
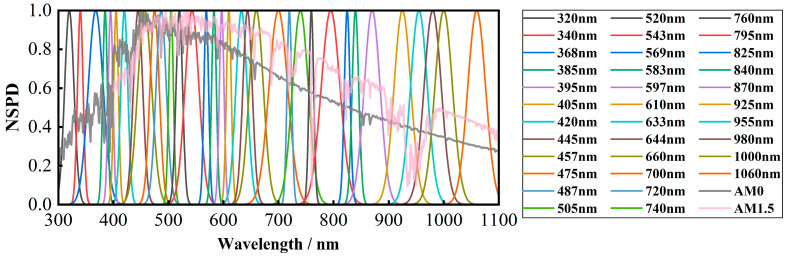
Normalized spectral power distribution of the selected 34 narrowband LEDs.

According to the available narrowband LED resources, 34 types of narrowband LEDs have been selected as the basic light sources. Their normalized spectral power distribution (NSPD) is illustrated in Figure 4. According to the IEC 60904-9:2020 international standard [34] and JJF 1615-2017 Calibration Specification for Solar Simulators [35], the instability of the solar simulator is less than 2% to meet the A class. After a systematic analysis, we require that the instability of the LED be less than 2%. However, changes in ambient temperature can affect LED performance. Elevated temperature leads to a decrease in optical output power, a shift in emission wavelength, and a shorter lifetime of LEDs, and there are differences in the temperature sensitivity of LEDs with different wavelengths. Therefore, system stability is ensured through optimized thermal design and precise LED drive current control.

**Figure 5:**
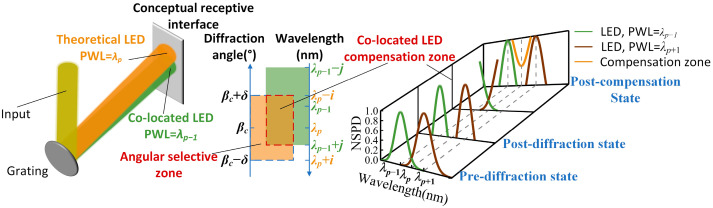
Principle of co-located LED compensation mapping.

According to Figure 4, the current selection of LEDs covering the spectral range of 1000 nm to 1100 nm is not optimal. The peak wavelength intervals between neighboring narrow-band LEDs are significant, which could lead to inferior spectral matching during the modulation process. To address this issue, based on Equation (1), a mapping relationship can be established between LEDs with similar spectral distributions and their positions in the spectral modulation optical engine. This setup aims to compensate for the deficiencies in spectral coverage. The strategy for this compensatory co-location mapping of LEDs is depicted in Figure 5, with the mapping relationship equation presented as follows:

(13) $$\beta =\arcsin(\sin\alpha -\frac{m\lambda }{d})$$

**Figure 6:**
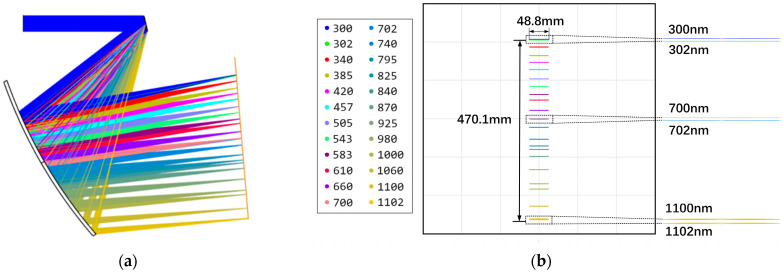
Design results of the diffraction beam-combining system. (**a**) System structure. (**b**) Localized point listings.

**Figure 7:**
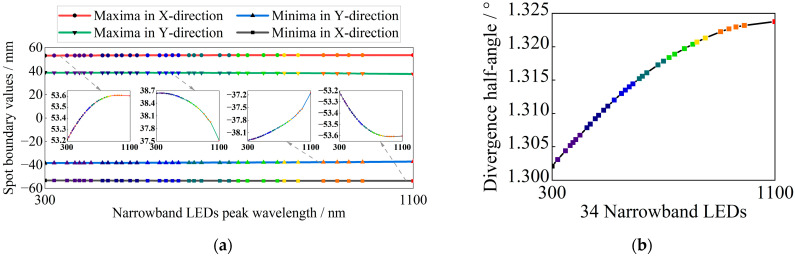
Narrowband LEDs combining beam efficiency: (**a**) spot boundary values of 34 narrowband LEDs in x-axis and y-axis directions; (**b**) divergence half-angle.

The imaging quality and spectral resolution of the diffraction beam combining system are crucial as they directly influence the emission angle and spot coincidence of each narrowband LED beam after spectral combining. These factors, in turn, affect the non-uniformity and angular diameter of the solar simulator. Given the selected narrowband LED peak wavelength interval of at least 10 nm and the packaging characteristics of the LED chip [36], the symmetric expansion of the spectral line width and asymmetric diffusion and expansion along the slit direction are controlled by optimizing the spherical aberration, comet aberration, and image dispersion of the diffractive combining system, so as to reduce the pointing offset of the sub-beams to improve alignment, and to reduce the divergence and blurring of the beam to improve the coincidence of the spot. The optimized diffraction combining system has a focusing mirror with a radius of curvature of 1554.45 mm, a grating with a groove density of 600 lp/mm, and a cylindrical lens with a sagittal focal length of 1380.00 mm. The spectral resolution is less than 2 nm across the 300–1100 nm band. It incorporates LED physical dimensions and spectral characteristics. Taking into account the physical dimensions and spectral characteristics of the LEDs, the divergence half-angle of the selected narrowband LEDs after combining them is less than 1.324°. The results of the diffraction beam-combining system design are illustrated in Figure 6, while the beam-combining efficiency of the selected narrowband LEDs is displayed in Figure 7.

As observed in Figure 7a, the light spot of each narrowband LED is distributed axisymmetrically about the x-axis. With an increase in peak wavelength, the spot radius expands from 53.21 mm to 53.60 mm. Conversely, the distribution on the upper and lower half-axes of the y-axis shows slight differences. As the peak wavelength increases, the radius of the upper half-axis decreases from 38.64 mm to 37.52 mm, and that of the lower half-axis decreases from 38.28 mm to 37.15 mm, according to the following equation:

(14) $$\mathrm{spot}\;\mathrm{coincidence}=\frac{{\left|r_{x}\right|}_{\min}\times {\left|r_{y}\right|}_{\min}}{{\left|r_{x}\right|}_{\max}\times {\left|r_{y}\right|}_{\max}}$$

**Figure 8:**
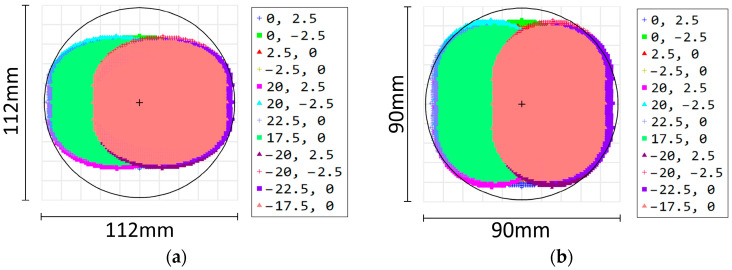
Comparison results of beam-combining efficiency; propagation at 800 mm from the last optical plane (**a**) without the cylindrical lens and (**b**) with the cylindrical lens.

The spot overlap of each narrowband LED is 95.42%, where ${\left|r_{x}\right|}_{\min}$ and ${\left|r_{y}\right|}_{\min}$ represent the minimum radius of each sub-spot on the x-axis and y-axis, respectively, and ${\left|r_{x}\right|}_{\max}$ and ${\left|r_{y}\right|}_{\max}$ represent the maximum radius, respectively. A comprehensive analysis of Figure 7a,b reveals that the divergence angle of the combining beam in the meridian direction is not consistent with that in the arc-vector direction, which introduces additional aberration when the combining beam directly enters the multi-aperture imaging-type reconstruction system. To address this issue, a cylindrical lens was added to the diffraction beam combining system. This adjustment reduced the radius in the arc-vector direction from 53.60 mm to 41.97 mm and decreased the divergence angle from 1.324° to 0.733°, which is slightly smaller than that in the meridional direction of 0.814°. The comparison results of the beam-combining efficiency are shown in Figure 8. Figure 8a shows the spot distribution characteristics of the central LED and the two edge LEDs at the maximum field of view without the cylindrical lens, while Figure 8b shows the corresponding results with the cylindrical lens.

### 3.2. Multi-Aperture Imaging Reconstruction System Optimization Design

**Figure 9:**
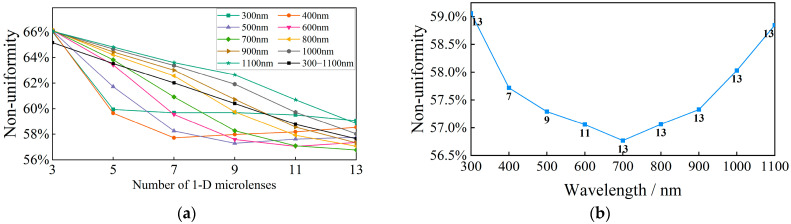

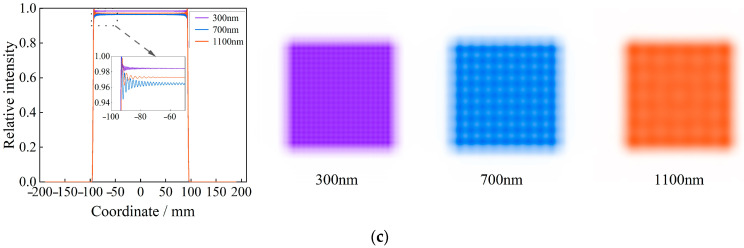
Beam reconstruction effect of multi-aperture imaging reconstruction system. (**a**) One-dimensional microlens number versus non-uniformity for single-wavelength and composite spectra; (**b**) distribution of single-wavelength minimum non-uniformity versus the number of one-dimensional microlenses; (**c**) one-dimensional relative intensity distribution and two-dimensional diffraction distribution at 300 nm, 700 nm, and 1100 nm.

The effectiveness of beam reconstruction in the solar simulator is primarily evaluated based on the non-uniformity and angular diameter of the irradiated area. Traditional evaluation methods using the Fresnel number assume a constant number of microlenses in the microlens array [37] and analyze only a single wavelength [38]. This approach does not fully address the practical needs of solar simulators to enhance the uniformity of the irradiation area through an increased number of microlenses, nor does it consider the non-uniformity across a composite spectral irradiation region. In response, this study proposes a method to maintain the F-number of the microlens constant, disregarding the aberration effects introduced by the lens. The 32′ angular diameter is used as the design parameter to determine the target surface spot size of the collimated integrating optics to be 190 mm × 190 mm, which is comparable to the international solar simulator spot size [39,40]. Building on this, the impact of the number of one-dimensional channels in the multi-aperture imaging reconstruction system on the non-uniformity of the reconstructed beam is analyzed for both single wavelength and composite spectra within the spectral range of 300 nm to 1100 nm. The results of this analysis are presented in Figure 9, based on Equations (11) and (12).

**Figure 10:**
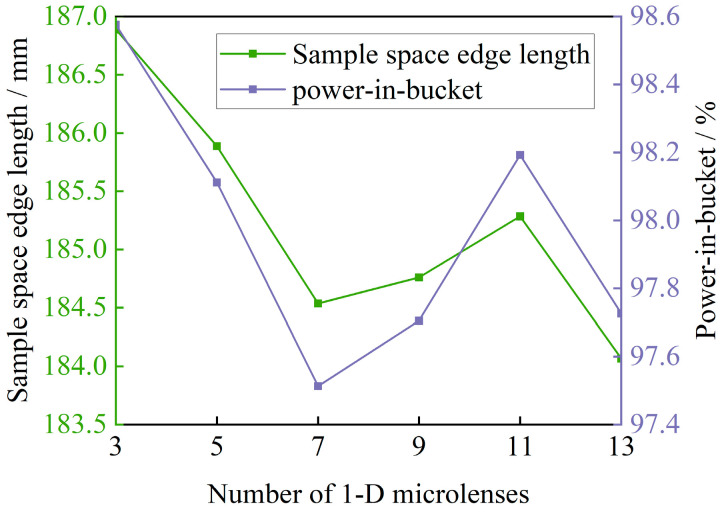
Power-in-bucket and sampling space edge length versus the number of channels in a multi-aperture imaging reconstruction system.

As shown in Figure 9a, increasing the number of channels substantially enhances the uniformity of the irradiated area. However, as the wavelength increases, the effectiveness of this strategy in reducing non-uniformity diminishes. Specifically, for wavelengths of 300 nm, 400 nm, 500 nm, and 600 nm, the non-uniformity converges to its smallest value when the number of channels is 5, 7, 9, and 11, respectively. Beyond these numbers, the correlation between the number of channels and non-uniformity becomes approximately positive. Conversely, for wavelengths between 800 nm and 1100 nm, a negative correlation is observed, and the trend of improvement in non-uniformity notably slows down as the number of channels increases. The non-uniformity of the composite spectra from 300 nm to 1100 nm shows a nearly linear negative correlation with the number of channels. Figure 9b further illustrates the specific distribution of the number of one-dimensional microlenses required to achieve the minimum non-uniformity at different wavelengths. While 300 nm achieves the smallest non-uniformity at 13 channels, the non-uniformity is similar for a number of channels ranging from 5 to 13. Thus, higher wavelengths can be inferred to require more channels to minimize non-uniformity, displaying an “U”-shaped relationship between wavelength and non-uniformity. In Figure 9c, the one-dimensional intensity distribution and two-dimensional diffraction distribution of single wavelengths are displayed for two maxima and one minimum of non-uniformity. The fringe diffraction effect significantly influences the non-uniformity, suggesting that the overall effectiveness of direct solar radiation simulation could be enhanced by selectively reducing the irradiation area on the target surface. This reduction excludes parts of the low-quality irradiation area, thereby improving the overall simulation effect. However, reducing the irradiated region also leads to energy loss in the solar simulator. To address this, the concept of “power-in-bucket” [41] is introduced, which represents the percentage of energy within the selected irradiated region relative to the total energy on the target surface. This approach ensures higher energy utilization with reduced non-uniformity. Figure 10 provides the relationship between power-in-bucket and the spatial edge length of the irradiated area sampling versus the number of channels of the multi-aperture imaging reconstruction system for the composite spectrum from 300 nm to 1100 nm with 1% non-uniformity.

**Figure 11:**
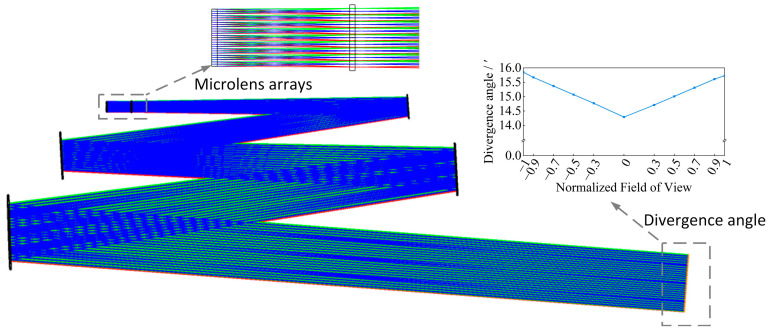
Design result of multi-aperture imaging reconstruction system.

As observed in Figure 10, adjusting the sampling space edge length from 189.94 mm to a range between 184.07 mm and 186.89 mm maintains the power-in-bucket between 97.51% and 98.58%. Simultaneously, non-uniformity within the sampling space is reduced to less than 1%. This demonstrates that significantly improved uniformity of the irradiation area can be achieved by excluding a minimal portion of energy affected by edge diffraction. Based on a comprehensive analysis of Figure 9 and Figure 10 and considering the high spectral radiance illumination near 500 nm within the solar spectrum, this study selects a 9 × 9 microlens array for optimal configuration. Subsequently, the target sampling interval is established at 184 mm. With a 32′ angular diameter as the target parameter, the multi-aperture imaging reconstruction system is optimally designed with the focal length of 132.98 mm for the sublenses and 6121.02 mm for the collimating integrating optic. The results of this design are shown in Figure 11, illustrating an effectively optimized approach to solar simulation.

## 4. Solar Direct Radiation Simulation and Reproduction Effect Analysis

The relative error of simulations conducted using the Monte Carlo ray tracing method remains within 2% when the number of rays emitted by each narrowband LED reaches 2 million [15]. Increasing the number of rays from 2 to 5 million yields only marginal improvements in simulation accuracy. Therefore, this study aims to verify the angular diameter, radiant illuminance, non-uniformity, and spectral matching degree of the simulation and reproduction effects. Additionally, this paper evaluates the direct solar radiation simulation and reproduction effects according to the IEC 60904-9:2020 international standard [34] and JJF 1615-2017 Calibration Specification for Solar Simulators [35].

### 4.1. Angle Diameter

**Figure 12:**
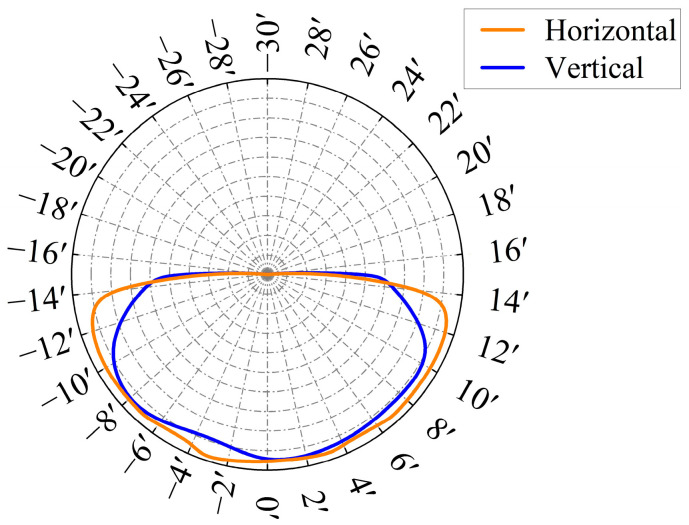
Solar angular diameter simulation results.

The horizontal angular diameter of the direct solar radiation simulation measures 31.6′, and the vertical angular diameter is 31.7′. Both values are within the specified solar angular diameter of 32′ ± 0.5′. The simulation results are depicted in Figure 12.

### 4.2. Spectral Matching Degree

The standard specifies that the spectral matching degree of solar simulators should be assessed based on the percentage of interval energy to determine the grade of spectral matching. However, this standard does not adequately reflect spectral deviations at specific wavelengths. To address this limitation, this study introduces an additional evaluation criterion that focuses on the relative error of spectral deviation. This new criterion quantitatively describes the degree of spectral deviation by calculating the relative error of the spectral distribution energy between the simulated spectral curves and the target spectral curves at the corresponding wavelengths. The calculation is performed using the following formula:

(15) $$\Delta E(\lambda )=\frac{E_{Sim}(\lambda )-E_{Tgt}(\lambda )}{E_{Tgt}(\lambda )}\times 100\%$$

where $E_{Sim}(\lambda )$ denotes the energy distribution of the simulated spectrum at wavelength λ, $E_{Tgt}(\lambda )$ denotes the energy distribution of the target spectrum at wavelength λ, and ΔE(λ) denotes the relative error between the simulated spectrum and the target spectrum at wavelength λ.

**Figure 13:**
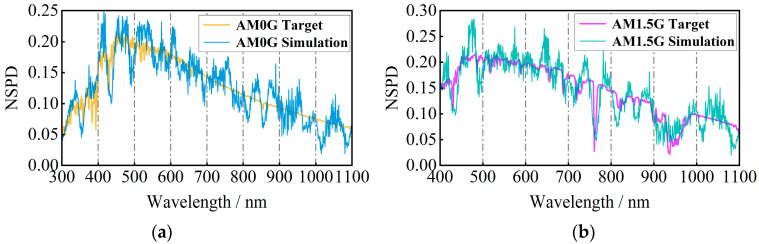

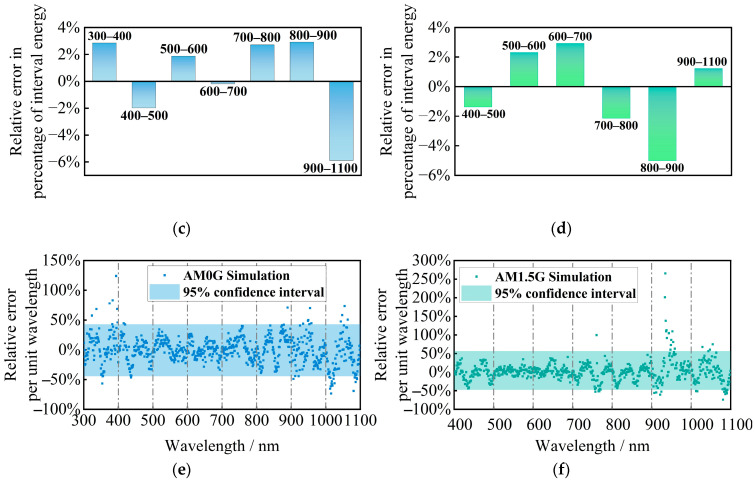
AM0G and AM1.5G simulation results. (**a**) Normalized spectral power distribution for AM0G spectral simulation (**b**) and AM1.5G. (**c**) Relative error in percentage of interval energy for AM0G (**d**) and AM1.5G. (**e**) Relative error per unit wavelength for AM0G (**f**) and AM1.5G.

To achieve a spectral matching degree that meets the A+ grade criteria of the percentage of interval energy (better than ±12.5%), the narrowband LED light sources are modulated within the spectral modulation optical engine. The simulation and reproduction results for the AM0G and AM1.5G solar spectra are depicted in Figure 13.

According to Figure 13, the maximum matching relative errors of the inter-area energy percentages for AM0G and AM1.5G are 5.89% and −5.01%, respectively, indicating that both have achieved an A+ grade. Additionally, the mean relative errors of the single band spectral simulations for AM0G and AM1.5G are −0.41% and 1.49%, respectively. The 95% confidence intervals for these simulations are −44.38% to 42.64% for AM0G, and 47.82% to 56.09% for AM1.5G.

### 4.3. Radiation Illumination and Non-Uniformity

**Figure 14:**
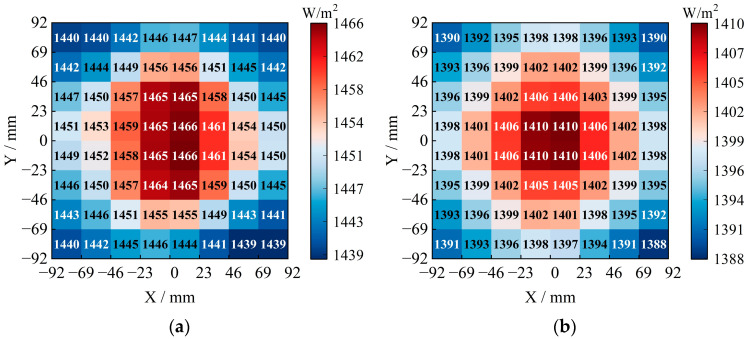
Radiation illuminance distribution: (**a**) AM0G; (**b**) AM1.5G.

According to the standard, the sampling area should constitute at least 80% of the designated test area, and the number of measurement points should be no fewer than 64. In this study, based on the results analyzed in Section 3.2, we divided 64 sampling areas within the 184 mm side-length sampling area, which constitutes 94.06% of the irradiated area. This division was made to achieve more detailed and accurate irradiation distributions. The average radiant illuminances for both AM0G and AM1.5G exceed the solar constants, measuring 1450.54W/m^2^ and 1398.80W/m^2^, respectively. The non-uniformities are recorded at 0.95% and 0.77%, respectively, meeting the A+ grade standards of the international guideline. These results are depicted in Figure 14.

### 4.4. Discussion

#### 4.4.1. Relative Error Correlation of Spectral Simulation Across Bands

Given that the spectral adjustment of the employed narrowband LEDs may involve operations across band intervals, this inevitably leads to interactions in the spectral simulation matching between different bands. Specifically, the spectral adjustment of a particular band may negatively or positively affect the matching accuracy of neighboring bands, thereby affecting the consistency and accuracy of the overall spectral simulation.

**Figure 15:**
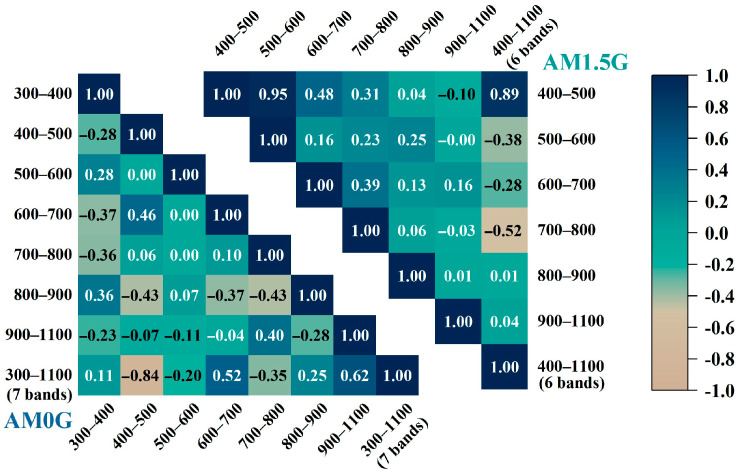
Relative error correlation coefficients for spectral simulations in each band interval for AM0G and AM1.5G.

To quantify the interaction between different spectral bands, a correlation analysis was used [42]. The relative error of the spectral simulation per unit wavelength within each band interval was defined as a set of random variables for the degree of spectral matching across all band intervals. From this, the Pearson correlation coefficient (PCC) was calculated [43]. The formula is as follows:

(16) $$r_{ij}=\frac{\sum \left(X_{i_{u}}-{\bar{X}}_{i})(X_{j_{u}}-{\bar{X}}_{j}\right)}{\sqrt{\sum {\left(X_{i_{u}}-{\bar{X}}_{i}\right)}^{2}\sum {\left(X_{j_{u}}-{\bar{X}}_{j}\right)}^{2}}}$$

where $r_{ij}$ denotes the spectral simulation relative error correlation coefficient between the *i*-th band interval and the *j*-th band interval, $X_{i_{u}}$ (or $X_{j_{u}}$) denotes the *u*-th unit-wavelength spectral simulation relative error in the *i*-th (or *j*-th) band interval, and ${\bar{X}}_{i}$ (or ${\bar{X}}_{j}$) denotes the average of the unit-wavelength spectral simulation relative error in the *i*-th (or *j*-th) band interval. The relative error correlation coefficients of the spectral simulation for each band interval are derived from the spectral simulation results of AM0G and AM1.5G in Figure 13, as shown in Figure 15.

As shown in Figure 15, when simulating and reproducing AM0G, the correlation coefficients between the 500–600 nm band and the neighboring bands of 400–500 nm and 600–700 nm are zero. The correlation coefficients with the other bands drop to a non-significant level, with the maximum correlation coefficient being only 0.28. This indicates that the 500 nm–600 nm bands have significant independence with respect to the simulation relative errors. In contrast, the correlation coefficient between the simulated AM1.5G solar spectrum in the 400–500 nm band and the 500–600 nm band is as high as 0.95. This significant positive correlation points to a strong positive linkage effect between the two bands. These quantitative correlation values provide a precise description for understanding the spectral simulation relative errors between different bands. They also offer a new perspective for revealing the intrinsic interrelationships between the bands. In particular, when there is a high degree of correlation between two bands, the trend of one band may become an effective indicator for predicting the change in the other band. This is of significant scientific importance and practical value for improving the accuracy of spectral simulation.

#### 4.4.2. Spectral Inconsistency

**Figure 16:**
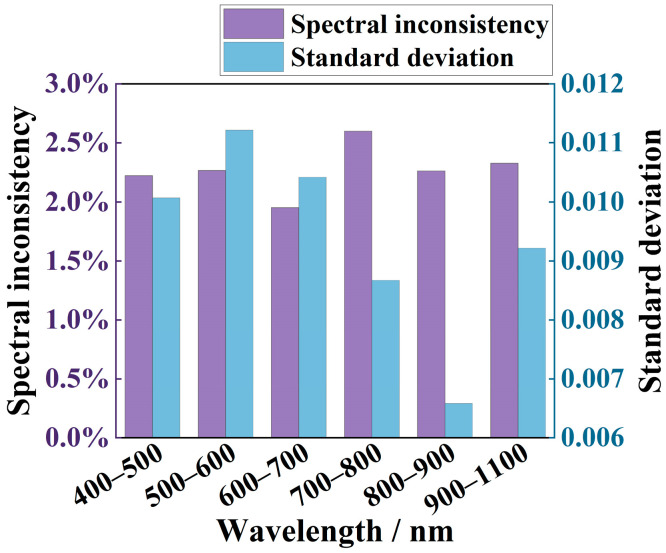
Spectral inconsistency of AM1.5G and its standard deviation.

In the performance evaluation of PV cells, the non-uniformity of the spectral distribution at different locations in the irradiated area can lead to differences in the response of PV cells to incident light. This may introduce deviations between the actual performance of PV cells and their test data in the laboratory [44], thus affecting the accurate evaluation of PV cell performance. To quantitatively analyze and evaluate this problem, the concept of spectral inconsistency is introduced in this paper. This allows for a more accurate quantification of the degree of AM1.5G matching at different locations, providing more accurate experimental data for PV cell performance tests. The spectral inconsistency evaluation formula is as follows:

(17) $$\chi_{i}=\frac{E_{i_{\max}}-E_{i_{\min}}}{E_{i_{\max}}+E_{i_{\min}}}\times 100\%$$

where $\chi_{i}$ denotes the spectral inconsistency in the ith band interval, $E_{i_{\max}}$ denotes the maximum radiant illuminance in the sampling points in the ith band interval, and $E_{i_{\min}}$ is the minimum radiant illuminance at the sampling points in the corresponding band. The AM1.5G spectral inconsistency was obtained by selecting the same 64 sampling regions as in the evaluation of radiance non-uniformity, as shown in Figure 16.

According to the results shown in Figure 16, the simulated spectra of AM1.5G exhibit significant consistency in the 400–1100 nm spectral range. Although the maximum value of spectral inconsistency is observed in the 700–800 nm band, it is only 2.60%, indicating high stability of the spectra even in the band with the worst consistency. Additionally, the largest standard deviation was recorded in the 500–600 nm band, with a value of 0.0112, further confirming significant spectral consistency. Overall, the results indicate that spectral differences are minimal and variability is maintained at a low level within the irradiated area, thus confirming the high stability of the spectral distribution.

#### 4.4.3. Effect of Defocus Distance and Tilt Angle on Non-Uniformity

**Figure 17:**
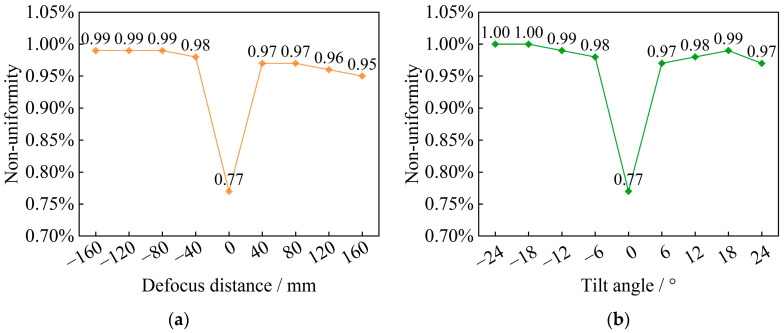
Effect of defocus distance and tilt angle on non-uniformity (**a**) defocus distance and (**b**) tilt angle.

When performing direct solar radiation tests on PV modules, the sun’s apparent motion trajectory within the equatorial latitude of ±23°27′ must be considered [45]. Therefore, to comprehensively assess the performance of PV modules, tests must be conducted at different solar irradiation angles to deeply analyze the performance of PV modules under different operating conditions. In addition, the radiant illuminance and non-uniformity of the direct solar radiation simulation may be affected by the defocusing distance and tilt angle. Therefore, this study analyzes the effect of the variation in the defocusing distance and tilt angle on non-uniformity, with a boundary condition of 1% non-uniformity in the case of AM1.5G spectral simulation. The results are shown in Figure 17.

As shown in Figure 17a, in the range of defocusing from −150 mm to 150 mm, the maximum value of non-uniformity is merely 0.99%, which satisfies the international standard A+ grade. As shown in Figure 17b, in the range of tilt angle from −24° to 24°, the maximum value of non-uniformity is 1.00%, which still meets the international standard A+ grade. The results show that despite a certain amount of defocusing and changes in the tilt angle, the direct solar radiation simulation system is still able to maintain a high degree of uniformity of irradiation area, which provides an accurate test environment for the performance evaluation of photovoltaic modules.

### 4.5. Comparison of Related Studies

Table 1 details the key performance metrics of solar simulators reported in the literature in recent years. These metrics include parameters such as irradiated area size, average radiant illuminance, non-uniformity, spectral matching, and angular diameter. The table also compares these metrics with those of the direct solar radiation simulation proposed in this study.

Compared with related studies, at the system architecture level, the optical system for the direct solar radiation simulation in this study consists of three modules, which is equivalent in complexity to existing xenon, metal halide, and LED solar simulators (all of which have optical structures consisting of multiple modules).

At the performance indicator level, the angular diameter in this study is 31.7′, which is smaller than the solar angular diameter of 32′. The two solar spectral simulation results, AM0G and AM1.5G, satisfy the requirements of the international standard A+ grade. The radiant illuminations under the two simulation conditions are 1450.54 W/m^2^ and 1398.80 W/m^2^, both exceeding one solar constant. The non-uniformities are 0.95% and 0.77%, respectively, meeting the international standard A+ grade.

In summary, compared with related studies, this study presents the only solar simulator in the world that can achieve one solar constant with an angular diameter of less than 32′. It is at the international leading level in terms of non-uniformity and spectral matching. Furthermore, no other relevant studies have been found that can simultaneously simulate the AM0G and AM1.5G solar spectra.

## 5. Conclusions

According to current research reports, no solar simulator can closely match natural direct solar radiation regarding key performance indicators, such as radiant illuminance, angular diameter, non-uniformity, and spectral matching. To achieve accurate indoor simulation and reproduction of direct solar radiation, this study has reached the following conclusions.

1. We have developed a direct solar radiation simulation and reproduction method, which utilizes the combination of multiple LED arrays and the grating anomalous dispersion principle to segment, collimate, and reconstruct the small-angle combined beam after diffraction combination via a multi-aperture imaging reconstruction system. The optical system for direct solar radiation simulation has been optimized and designed, eliminating the disadvantage of low energy utilization caused by controlling the angular diameter using the small-aperture imaging principle.
2. We have established an optical model for solar spectrum reconstruction and defined the precise mapping relationship between the peak wavelengths of narrowband LEDs and their spatial positions. This provided the basis for designing a multi-aperture imaging reconstruction system according to the principle of beam reconstruction optics. Based on scalar diffraction theory, we completed the mathematical characterization of the diffraction energy distribution of the reconstructed beam on the target surface in the solar spectral range.
3. We simulated and analyzed the angular diameter, degree of spectral matching, radiant illuminance, and non-uniformity of the designed direct solar radiation simulator. The results show that the angular diameter is 31.7′, meeting the simulation accuracy of the solar angular diameter of 32′ ± 0.5′. The degree of spectral matching for AM0G and AM1.5G reached the international standard A+ class. The radiant illuminance under the simulation conditions of the two spectra is 1450.54 W/m^2^ and 1398.80 W/m^2^, respectively, both exceeding the solar constant. The non-uniformities are 0.95% and 0.77%, both meeting the international standard A+ class.
4. Using Pearson’s correlation coefficient, we quantitatively analyzed the correlation of the relative errors of spectral simulation in each band interval in the spectral simulation cases of AM0G and AM1.5G. This analysis provides a precise description of the relationship between the relative errors of spectral simulation across different bands. For the special requirements of AM1.5G simulation in photovoltaic cell performance evaluation, we analyzed the spectral inconsistency in the simulated AM1.5G spectral case and the effects of defocus distance and tilt angle on non-uniformity. We found that the non-uniformity of the designed solar simulator, within the ranges of defocusing of −150 mm to 150 mm and tilt angle of −24° to 24°, still meets the A+ grade.

The direct solar radiation simulator developed in this study provides significant advantages for high-precision applications such as satellite attitude component calibration and aerospace solar panel performance testing. Although the current design is primarily intended for laboratory environments, the proposed system architecture supports illumination area scaling through optical path redesign. For large-scale applications, the architecture facilitates modular integration to accommodate diverse testing requirements. We plan to develop a physical prototype of the proposed system and conduct experimental tests on irradiance, spectral matching, non-uniformity, and angular diameter, with the results compared against simulation data. In addition, by increasing the variety of narrowband LEDs, we can enrich spectral simulation types while improving spectral matching accuracy. We will also consider the risks associated with possible mechanical interference, temperature or vibration misalignment of the optical system, and possible changes in the characteristics of the components during long-term operation. This study provides important technical support for future solar energy conversion, storage, and applications, ultimately supporting the development, optimization, and evaluation of photovoltaic products.

## Figures and Tables

**Table 1:** Comparison of relevant studies in domestic and international research.

Relevant Studies	Irradiation Area (mm × mm)	Average Irradiance (w·m^−2^)	Non-Uniformity (%)	Angular Diameter	Spectral Range (nm)	Spectral Matching Class	System Architecture
Jian Jin et al., 2019 [12]	4000 × 3000	940	8	2.6°	-	-	Lamp array of 7 radiant modules, Optical integrator, Collimating lens
Leopoldo Martínez-Manuel et al., 2021 [15]	2000 × 1000	1198	1.4	-	-	-	Multi-lamp array of 26 subunits. Each subunit contains a 575 We metal halide lamp and a parabolic reflector
Mehdi Tavakoli et al., 2021 [25]	23 × 23	1000	A Class	-	250–1000	A Class (AM1.5G)	19 types of LED and total internal reflector
Al-Ahmad et al., 2022 [27]	320 × 200	1000	1.7	-	350–1100	A Class (AM1.5G)	LED source plane array(266 LEDs of ten colors) and shaping components of the mirrored light housing and diffuser
Chao Sun et al., 2022 [28]	50×50	1000	3.2	±3°	400–1100	A Class (AM1.5G)	Light source system, two total reflection panels, field lens
Zhiqiang Du et al., 2023 [29]	1000 × 1000	726	15.74	-	350–2500	-	metal halide lamps, halogen lamps, high power white LEDs and 31different kinds of monochromatic LEDs
This study	184 × 184	1450.54	0.95 (A+Class)	31.7′	300–1100	A+Class (AM0G)	Spectrally modulated optical engine, diffraction combining system, and multi-aperture imaging reconstruction system
		1398.80	0.77 (A+Class)		400–1100	A+Class (AM1.5G)	

## Data Availability

The data presented in this study are available upon request from the corresponding author.

## References

[1] Yeritsyan H.N., Sahakyan A.A., Grigoryan N.E., Harutyunyan V.V., Arzumanyan V.V., Tsakanov V.M., Grigoryan B.A., Davtyan H.D., Dekhtiarov V.S., Rhodes C.J. (2021). Space low earth orbit environment simulator for ground testing materials and devices. Acta Astronaut..

[2] Verduci R., Romano V., Brunetti G., Yaghoobi Nia N., Di Carlo A., D’Angelo G., Ciminelli C. (2022). Solar energy in space applications: Review and technology perspectives. Adv. Energy Mater..

[3] Smestad G.P., Krebs F.C., Lampert C.M., Granqvist C.G., Chopra K.L., Mathew X., Takakura H. (2008). Reporting solar cell efficiencies in solar energy materials and solar cells. Sol. Energy Mater. Sol. Cells.

[4] Garnier B.J., Ohmura A. (1970). The evaluation of surface variations in solar radiation income. Sol. Energy.

[5] Zhang Y., Yi H., Iraqi A., Kingsley J., Buckley A., Wang T., Lidzey D.G. (2017). Comparative indoor and outdoor stability measurements of polymer based solar cells. Sci. Rep..

[6] Vallerotto G., Martín F., Macías J., Herrero R., San José L.J., Askins S., Núñez R., Domínguez C., Antón I. (2023). Collimated solar simulator for curved PV modules characterization. Sol. Energy Mater. Sol. Cells.

[7] Liu S., Zhang G., Sun G., Wang L., Gao Y. (2017). Design of an optical system for a solar simulator with high collimation degree and high irradiance. J. Opt. Technol..

[8] Gao Y., Zhu X., Chen J., Xie Y., Hong J., Jin J., Han J., Zhang X., Xu C., Zhang Y. (2024). Constructing the large-scale collimating solar simulator with a light half-divergence angle < 1° using only collimating radiation modules. Renew. Energy.

[9] Wang J., Qiu Y., Li Q. (2023). A high-performance solar simulator pursuing high flux and fine uniformity: Modelling, optimization, and experiment. Sol. Energy.

[10] Rapp C., Straub V., Wet van Rooyen D., Thor W.Y., Siefer G., Bett A.W. (2015). Optical investigation of a sun simulator for concentrator PV applications. Opt. Express.

[11] Dong X., Sun Z., Nathan G.J., Ashman P.J., Gu D. (2015). Time-resolved spectra of solar simulators employing metal halide and xenon arc lamps. Sol. Energy.

[12] Jin J., Hao Y., Jin H. (2019). A universal solar simulator for focused and quasi-collimated beams. Appl. Energy.

[13] Meftah M., Damé L., Bolsée D., Hauchecorne A., Pereira N., Sluse D., Cessateur G., Irbah A., Bureau J., Weber M. (2018). SOLAR-ISS: A new reference spectrum based on SOLAR/SOLSPEC observations. Astron. Astro Phys..

[14] Zhu Q., Xuan Y., Liu X., Yang L., Lian W., Zhang J. (2020). A 130 kWe solar simulator with tunable ultra-high flux and characterization using direct multiple lamps mapping. Appl. Energy.

[15] Martínez-Manuel L., Wang W., Peña-Cruz M. (2021). Optimization of the radiative flux uniformity of a modular solar simulator to improve solar technology qualification testing. Sustain. Energy Technol. Assess..

[16] Hamady M., Lister G.G., Zissis G. (2016). Calculations of visible radiation in electrodeless HID lamps. Lighting Res. Technol..

[17] Liu Y., Liu M., Yang H., Yi Z., Zhang H., Tang C., Deng J., Wang J., Li B. (2025). Photoelectric simulation of perovskite solar cells based on two inverted pyramid structures. Phys. Lett. A.

[18] Ren J., Ma Q., Sun X., Wang S., Liu G., Yang H. (2025). Interface-engineering enhanced photocatalytic conversion of CO2 into solar fuels over S-type Co-Bi2WO6@Ce-MOF heterostructured photocatalysts. J. Colloid Interface Sci..

[19] Liu Z., Zhang J., Rao G., Peng Z., Huang Y., Arnold S., Liu B., Deng C., Li C., Li H. (2024). Accelerating Photostability Evaluation of Perovskite Films through Intelligent Spectral Learning-Based Early Diagnosis. ACS Energy Lett..

[20] Kabir M.Z. (2024). Analytical Model for Current–Voltage Characteristics in Perovskite Solar Cells Incorporating Bulk and Surface Recombination. Micromachines.

[21] Zeng L.R., Ding B., Zhang G., Liu Y., Zhang X., Yang G.J., Chen B. (2024). Elimination of buried interfacial voids for efficient perovskite solar cells. Nano Energy.

[22] Li X., Aftab S., Mukhtar M., Kabir F., Khan M.F., Hegazy H.H., Akman E. (2025). Exploring Nanoscale Perovskite Materials for Next-Generation Photodetectors: A Comprehensive Review and Future Directions. Nano-Micro Lett..

[23] Mutlugun E., Soganci I.M., Demir H.V. (2008). Photovoltaic nanocrystal scintillators hybridized on Si solar cells for enhanced conversion efficiency in UV. Opt. Express.

[24] Barbet A., Paul A., Gallinelli T., Balembois F., Blanchot J.P., Forget S., Chénais S., Druon F., Georges P. (2016). Light-emitting diode pumped luminescent concentrators: A new opportunity for low-cost solid-state lasers. Optica.

[25] Tavakoli M., Jahantigh F., Zarookian H. (2021). Adjustable high-power-LED solar simulator with extended spectrum in UV region. Sol. Energy.

[26] Al-Ahmad A., Clark D., Holdsworth J., Vaughan B., Belcher W., Dastoor P. (2022). An economic LED solar simulator design. IEEE J. Photovolt..

[27] Al-Ahmad A., Holdsworth J., Vaughan B., Belcher W., Zhou X., Dastoor P. (2022). Optimizing the Spatial Nonuniformity of Irradiance in a Large-Area LED Solar Simulator. Energies.

[28] Sun C., Jin Z., Song Y., Chen Y., Xiong D., Lan K., Huang Y., Zhang M. (2022). LED-based solar simulator for terrestrial solar spectra and orientations. Sol. Energy.

[29] Du Z., Zhao H., Jia G., Li X. (2023). Design, fabrication, and evaluation of a large-area hybrid solar simulator for remote sensing applications. Opt. Express.

[30] Fontenla J., White O.R., Fox P.A., Avrett E.H., Kurucz R.L. (1999). Calculation of solar irradiances. I. Synthesis of the solar spectrum. Astrophys. J..

[31] Fryc I., Brown S.W., Ohno Y. Spectral matching with an LED-based spectrally tunable light source. Proceedings of the Fifth International Conference on Solid State Lighting.

[32] Voelkel R., Weible K.J. Laser beam homogenizing: Limitations and constraints. Proceedings of the Optical Fabrication, Testing, and Metrology III.

[33] Buttner A., Zeitner U.D. (2002). Wave optical analysis of light-emitting diode beam shaping using microlens arrays. Opt. Eng..

[34] (2020). 2020-Photovoltaic Devices–Part 9: Classification of Solar Simulator Characteristics.

[35] (2017). Calibration Specification for Solar Simulators.

[36] Sun C.C., Chien W.T., Moreno I., Hsieh C.C., Lo Y.C. (2009). Analysis of the far-field region of LEDs. Opt. Express.

[37] Zimmermann M., Lindlein N., Voelkel R., Weible K.J. Microlens laser beam homogenizer: From theory to application. Proceedings of the Laser Beam Shaping VIII.

[38] Du Z. (2017). The Research of Solar Simulator Theory and Application. Ph.D. Thesis.

[39] Wang J., Qiu Y., Li Q., Xu M., Wei X. (2021). Design and experimental study of a 30 kWe adjustable solar simulator delivering high and uniform flux. Appl. Therm. Eng..

[40] Wang W., Aichmayer L., Garrido J., Laumert B. (2017). Development of a Fresnel lens based high-flux solar simulator. Sol. Energy.

[41] Wu C., Ko J., Davis C.C. (2016). Plenoptic mapping for imaging and retrieval of the complex field amplitude of a laser beam. Opt. Express.

[42] Godfrey K.R. (1980). Correlation methods. Automatica.

[43] Zhou H., Deng Z., Xia Y., Fu M. (2016). A new sampling method in particle filter based on Pearson correlation coefficient. Neurocomputing.

[44] Minemoto T., Toda M., Nagae S., Gotoh M., Nakajima A., Yamamoto K., Takakura H., Hamakawa Y. (2007). Effect of spectral irradiance distribution on the outdoor performance of amorphous Si//thin-film crystalline Si stacked photovoltaic modules. Sol. Energy Mater. Sol. Cells.

[45] Geisemeyer I., Tucher N., Müller B., Steinkemper H., Hohl-Ebinger J., Schubert M.C. (2017). Angle dependence of solar cells and modules: The role of cell texturization. IEEE J. Photovolt..

